# Cannabis Use and Car Crashes: A Review

**DOI:** 10.3389/fpsyt.2021.643315

**Published:** 2021-05-28

**Authors:** Ulrich W. Preuss, Marilyn A. Huestis, Miriam Schneider, Derik Hermann, Beat Lutz, Alkomiet Hasan, Joseph Kambeitz, Jessica W. M. Wong, Eva Hoch

**Affiliations:** ^1^Vitos Klinik Psychiatrie und Psychotherapie, Herborn, Germany; ^2^Department of Psychiatry, Psychotherapy and Psychosomatics, Martin-Luther University Halle-Wittenberg, Halle, Germany; ^3^Institute on Emerging Health Professions, Thomas Jefferson University, Severna Park, MD, United States; ^4^Institute of Psychology, Heidelberg University, Department of Developmental and Biological Psychology, Heidelberg, Germany; ^5^Department of Addictive Behaviour and Addiction Medicine, Central Institute of Mental Health, Mannheim, Germany; ^6^Institute of Physiological Chemistry and German Center for Resilience, University Medical Center of the Johannes-Gutenberg-University Mainz, Mainz, Germany; ^7^Clinic and Polyclinic for Psychiatry and Psychotherapy, Clinic of the Ludwig-Maximilian-University Munich, Munich, Germany; ^8^Am Homberg Clinic, Bad Wildungen, Germany

**Keywords:** cannabis, cannabinoids, THC, automobile driving, impaired driving, driving safety, driving skills, driving ability

## Abstract

In this review, state-of-the-art evidence on the relationship between cannabis use, traffic crash risks, and driving safety were analyzed. Systematic reviews, meta-analyses, and other relevant papers published within the last decade were systematically searched and synthesized. Findings show that meta-analyses and culpability studies consistently indicate a slightly but significantly increased risk of crashes after acute cannabis use. These risks vary across included study type, crash severity, and method of substance application and measurement. Some studies show a significant correlation between high THC blood concentrations and car crash risk. Most studies do not support this relationship at lower THC concentrations. However, no scientifically supported clear cut-off concentration can be derived from these results. Further research is needed to determine dose-response effects on driving skills combined with measures of neuropsychological functioning related to driving skills and crash risk.

## Introduction

Cannabis is worldwide the most frequently used illicit drug (1). Rates of driving under the influence of cannabis rose in recent years (2). For instance, the DRUID (Driving Under the Influence of Drugs, Alcohol and Medicines) project reported on 50,000 drivers from 13 different countries of whom 1.32% used cannabis (3). On weekends, the rates of positively screened cannabis users in traffic were 10–12% and in subjects involved in a car crash 26–27% (3, 4), while 0.5–7.6% of persons involved were severely injured.

“Cannabis-impaired driving” describes the impairment caused by cognitive and psychomotor effects of Δ9-tetrahydrocannabinol (THC) which negatively influences a driver of a motor vehicle after THC consumption. In contrast, a “cannabis-positive driver” is someone driving a motor vehicle with any detectable THC concentration in blood, oral fluid, or urine with/without showing impairments in his driving. “Driving under the influence of cannabis” (DUIC) is a judicial term which refers to a driver exhibiting a measured reduction in cognitive or psychomotor skills in conjunction with a defined THC concentration in blood, oral fluid, or urine (5).

THC acutely and probably chronically reduces different cognitive and psychomotor abilities needed for driving, like balance, executive function, motor impulsivity and impulse control, perception, psychomotor speed, short-term memory, visual processing, and working memory (reaction time and accuracy) (3, 6). All these reductions may be dose-dependent (4), negatively influence driving skills and car crash risk, and may worsen with duration and frequency of cannabis use (5). However, results on the relationship between driving performance and traffic crash risk under the influence of cannabis revealed inconsistent findings (6–8).

Methodological heterogeneity may explain mixed findings. As summarized by Asbridge et al. (2), several study types need to be considered for a comprehensive evaluation of the relationship between cannabis use, driving skills, and car crash risk [e.g., sample surveys, laboratory experiments, and epidemiological studies like case-control and their variant, “culpability” studies (2, 7)]. Each of the study approaches has strengths and weaknesses. For instance, it is challenging in epidemiological studies to obtain the proportion of cannabis users in their samples. Some studies rely on self-reports which may underestimate the actual fraction of cannabis users. Only a minority of all studies assesses cannabis content in blood or other body tissues or fluids at the time of the crash. However, it may be unclear whether the cannabis consumption is occasional or frequent, or when the last cannabis intake occurred prior to the crash. Among other factors which may contribute to crash risk under the influence of cannabis are the use of additional substances (e.g., alcohol) and the frequency and chronicity of cannabis use. Again, only a fraction of studies reports a combined assessment of various legal and illicit substances or controls for other confounding factors (7).

Timing of cannabis use prior to an event and frequency of cannabis use have different effects on driving skills. While laboratory and experimental studies are often of small sample size, they usually assess specific driving impairment under various but defined doses of smoked or oral cannabis products (6) in an artificial environment with participants themselves aware of possible impairments from cannabis use who tried to compensate with slower and less risky driving (6).

These laboratory investigations are complemented by a recent double-blind, randomized clinical trial on *n* = 26 healthy occasional cannabis users (9) which were exposed to vaporized THC-dominant, CBD-dominant, THC/CBD-equivalent, and placebo cannabis. The end point measured was standard deviation of lateral position (SDLP; a measure of lane weaving) during 100 km on-road driving tests 40 and 240 min after cannabis consumption.

The SDLP following vaporized THC-dominant and THC/CBD-equivalent cannabis vs. placebo was shown to be significantly greater at 40–100 min but not 240–300 min, but there were no significant differences between CBD-dominant cannabis and placebo. The doses tested here may be representative of common usage.

In comparison, epidemiological and survey samples are frequently not assessed regarding their neuropsychological impairments which may lead to heterogeneity of study results. Not surprisingly, some studies which analyzed the association between use of cannabis and the risk of car vehicle crashes reported an increased risk (10–12), while others reached inconclusive results (13, 14).

Different study types have various outcome criteria. Some of the meta-analytical studies included crashes with injuries and fatalities (2) while others included simple collisions (6, 15), all of which have a different profile of risk factors. Also, five meta-analyses summarized the effect size of DUIC (2, 6, 15–17) and suggested that the risk of vehicle crashes is increased by cannabis. All studies used a random-effect model to assess the effect size of cannabis use on car crashes. However, all studies also reported a significant heterogeneity across analyzed studies. The more recent meta-analysis reported that the significant heterogeneity found was caused by publication bias favoring studies showing a positive association between DUIC and car crash risk (15).

In summary, one study approach alone may be insufficient to assess driving skills and traffic offenses in DUIC due to a variety of confounding and methodological factors.

## Research Questions

This review has three purposes. First, we searched the scientific evidence for a link between the use of cannabis, driving safety, and risk of car crashes by concentrating on recent meta-analyses and large case-control-studies. Second, we evaluated the role of sample subgroups (e.g., role of co-consumed alcohol), study types (e.g., case control vs. culpability), and kind of crash (collision, injury, fatality) regarding DUIC and car crash risk. Third, independent of meta-analyses, we wanted to clarify the relationship between car crashes and different concentrations of THC detected in blood or other body fluids of drivers.

## Method

This review is part of an expert report [Cannabis: Potential and Risks: A scientific analysis (CaPRis)] funded by the German Ministry of Health (18). It followed guidance of the Cochrane Collaboration (19). Other parts of this report have been published earlier (20). The study protocol can be found at: http://www.crd.york.ac.uk/PROSPERO/display_record.php?ID=CRD42016033249.

### Rationale

In accordance with the German Association of the Scientific Medical Societies (AWMF) (21) this review followed a top-down search strategy. We prioritized studies with the highest level of evidence, i.e., aggregated data in systematic reviews and meta-analyses according to PRISMA (22). If our first search failed to answer the clinical questions, we then included studies with a lower level of evidence (e.g., cohort studies, case control studies). PubMed, PsycINFO, Medline, Embase, and the Cochrane Library were systematically searched. References of the identified reviews and meta-analyses were manually searched to identify additional studies. Researchers in this field were contacted. Screening of the search results, assessing eligibility and methodological quality of full-text articles, data extraction, and data synthesis were independently performed by two reviewers; disagreements were resolved through consensus or referral to a third expert.

### Eligibility Criteria

The inclusion criteria were systematic reviews or meta-analyses and prospective cohort studies investigating the effects of cannabis use on cognition, intelligence, driving performance, and traffic crashes. All studies published in English or German from 2005 to 2020 were considered. Outcome criteria wasn't specified. Exclusion criteria were non-systematic reviews, reviews without documented systematic literature search, systematic reviews not focusing on cannabis/cannabinoids, animal and molecular studies, expert opinion, and position statements.

### Search Strategy and Methodological Assessment

For the global search, terms (MeSH-Terms) used were: “Cannabis OR cannabinoid^*^ OR hemp OR hanf” OR 2) “Mariuana OR Marihuana OR Marijuana.” Search strings were built, pilot-tested, and adopted to different databases. All studies included were rated for their methodological quality using the SIGN-checklist (23) revealing scores ranging from “high quality (++),” “acceptable quality (+),” “low quality (–),” to “unacceptable – reject.” Each study was rated on its level of evidence, based on study type and quality (24) ranging from “1” (highest level of evidence) to “5” (lowest level of evidence).

### Data Synthesis

This review applied a qualitative data synthesis approach. An aggregated data analysis couldn't be used because of the high heterogeneity of primary outcome measures. The study results were interpreted with respect to their sample size, level of evidence, risk of bias, and level of heterogeneity/homogeneity. If there was a case of duplicate primary studies, the following preference criteria was included (23): the availability of numerical data or results; the highest SIGN-rating (Quality assessment tool for systematic reviews); most recent date of publication; larger number of studies and observations. Assessments were made independently for each outcome. If two reviews with duplicate primary studies reported on different outcomes, both were eligible for inclusion.

## Results

**Figure 1:**
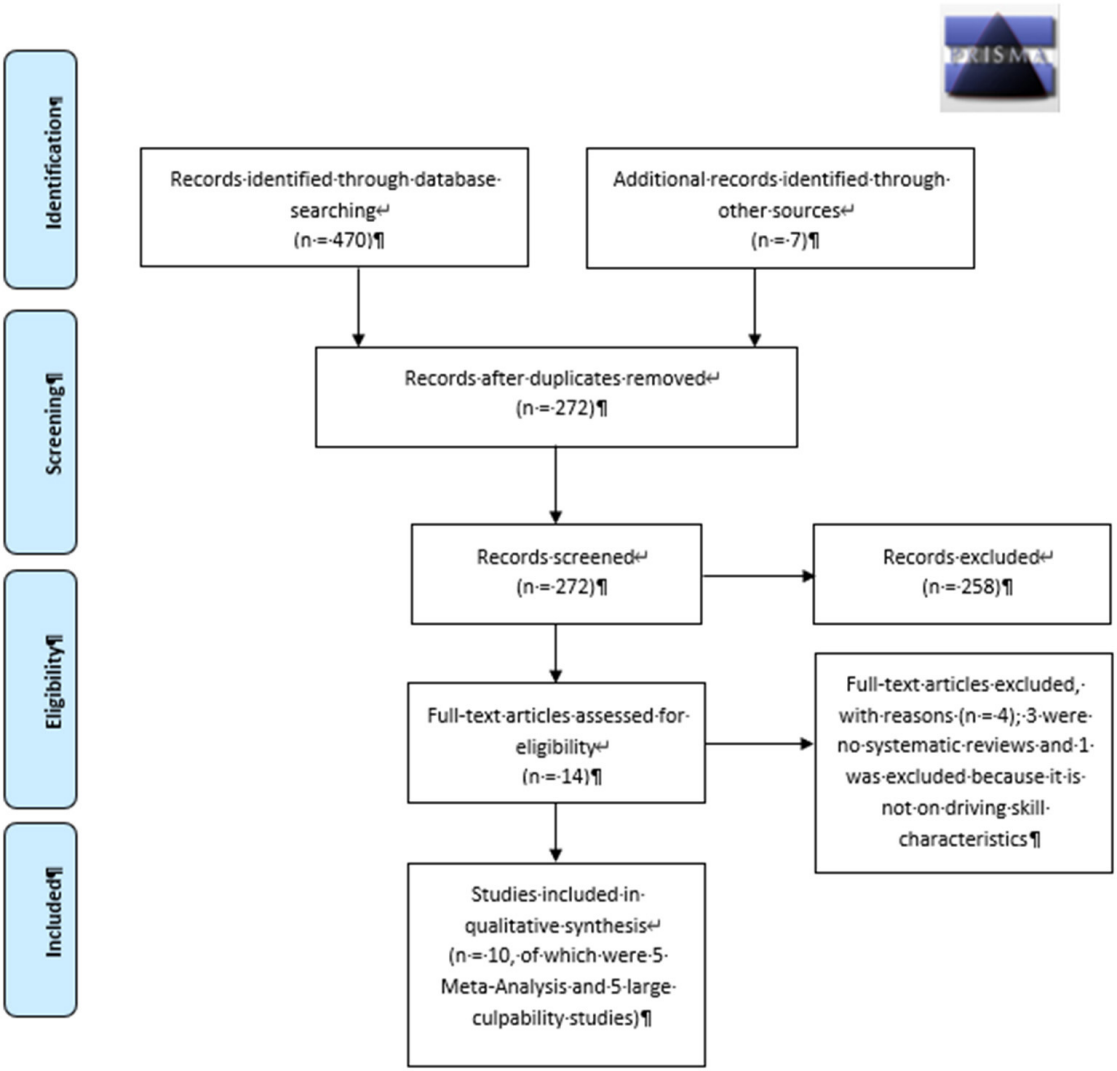
Prisma flow diagram cannabis use and car crash risk (see also Results section).

The global literature search identified 470 publications across all databases. After removal of duplicates, 272 manuscripts remained and were screened for eligibility (Figure 1). The literature search on cannabis, driving skills, and related crash risks resulted in 14 publications of which three were not systematic reviews (1, 3, 6) and one report was not included for further analyses (25) because it did not report on driving skill characteristics. Three studies (2, 7, 15) reported meta-analytic results on cannabis use and car accidents and were considered for inclusion. Two subsequent studies were published after the CAPRIS literature search (15, 17) and were also included. To evaluate the relationship between THC blood concentrations and crash risk, five large case-control (culpability) studies were included (26–30). According to the included study designs, the evidence was rated 3 as “good to moderate” quality (24).

### Results of Meta-Analyses

Asbridge et al. (2) conducted the first meta-analysis, which we rated as “high” for its methodological quality (“++”) according to SIGN (23). It included four studies on traffic accidents and injuries, and five studies on fatalities related to cannabinoid use. The authors used the Newcastle-Ottawa-Scale (31) for the assessment of retrieved studies. Their screenings resulted in a “high”-rating for four and a “moderate”-rating for five of these studies. In eight of the nine included studies, THC was analyzed in whole blood, serum, or plasma while one included study (10) relied on self-reported use. Seven of the nine included studies reported significantly increased traffic crash risk in individuals up to 1 h after cannabinoid use. Overall meta-analytical statistics demonstrated an OR of 1.92 (95% CI 1.35–2.73, Table 1) for crashes after cannabis use and thus almost doubled the risks compared to controls, after weighting the studies. Hence, heterogeneity of results and research designs across the nine studies were substantial. Studies which received “moderate” quality rating reported a lower car crash risk (OR of 1.78) compared to those with “high” quality ratings (OR 2.21). Furthermore, case-control studies had significantly higher OR for traffic crashes following cannabis use (OR = 2.79; 95% CI: 1.23–6.33) than culpability studies (OR = 1.65; 95% CI: 1.31–3.36, Table 1). Additional analyses revealed that traffic crash risk is increased in both fatal and non-fatal cases after cannabinoid consumption while fatal cases only achieved statistical significance (fatal crashes OR 2.1; 95% CI 1.31–3.36 vs. non-fatal crashes OR 1.74; 95% CI 0.88–3.46).

**Table 1:** Meta-analyses of driving under the influence of cannabis (DUIC) and risks of car vehicle crash studies.

**References**	***N* participants**	***N* studies**	**Odds ratio selected outcomes**	**95% CI**	**SIGN**
Hostiuc et al. (15)	*n* = 245,591	24 studies	REM:		++
			All: 1.89	1.58–2.26	
			Collision: 1.95	1.24–3.05	
			Injury: 2.16	1.41–3.28	
			Fatal: 1.73	1.36–2.19	
			Case-control: 1.95	1.51–2.51	
			Other studies: 1.81	1.38–2.39	
Rogeberg and Elvik (17)			REM		+
	*n* = 50,877 +	9 + 9 studies	All: 1.36	1.15–1.61	
	*n* = 93,229	21 studies	Fatal 1.32	1.08–1.62	
	*n* = 239,739	(re-analysis)	Case Control 1.60	1.19–2.15	
Elvik (7)		27 studies	REM		++
			Property damage: 1.26	1.10–1.44	
			Injury: 1.10	0.88–1.39	
			Fatal 1.26	0.88–1.81	
			Self-report:1.31	0.80–2.15	
			Lab Tests: 1.16	0.79–1.71	
Li et al. (16)	*n* = 93,200	9 studies	REM		+
			All: 2.66	2.07–3.41	
			Case-control: 2.63	1.87–3.71	
			Cohort: 2.04	1.36–3.07	
			Cross-sectional: 3.61	2.37–5.49	
			Self-report: 2.93	2.07–4.17	
			Blood/urine test: 2.26	1.46–3.49	
Asbridge et al. (2)	*n* = 51,783	9 studies	REM		++
			All: 1.92	1.35–2.73	
			Case-control: 2.79	1.23–6.33	
			Culpability: 1.65	1.11–2.46	
			Fatal: 2.10	1.31–3.36	
			Non-fatal: 1.74	0.88–3.46	

*CI, confidence interval; SIGN Methology Checklist: High quality (++), acceptable (+), unacceptable (–), reject (0); REM, random effects model*.

The meta-analysis of Li et al. (16) received an “acceptable” (“+”) quality rating according to SIGN (23). Nine studies were included in their analyses of which six investigated non-fatal and one fatal car crashes while two investigated both fatal and non-fatal car crashes under the influence of cannabis. Study types considered in this meta-analysis included case-control, cross-sectional, and cohort studies. Five of the nine studies assessed marijuana use based on self-reported data, and 2 were based on urine, one on blood, and one on both urine and blood tests. Eight of the nine studies reported an increased traffic crash risk after cannabis use except for one report from Thailand (32). Results of the meta-analysis demonstrated a statistically significant OR of 2.66 (95% CI 2.07–3.41, Table 1) after weighting study results. Most results stayed significant after controlling for several confounding factors like alcohol intake, while adjusted ORs decreased after statistical correction. However, heterogeneity of results also achieved statistical significance. Secondary analyses revealed that study designs, methods of drug testing, or age of study participants influenced outcomes. It is important to note that studies had variable approaches to assess cannabis use before driving. While cross-sectional and case-control studies limited cannabis use by self-report and laboratory tests between 1 and 3 h before driving, cohort studies relied on self-reports of current and last year's cannabis use. Further, in their analyses, OR for several potentially confounding factors were determined. Cross-sectional studies (two studies) had the highest crash risk (OR 3.61), followed by case control-studies (five studies) (OR 2.63) and cohort studies (two studies, OR 2.04). Estimated ORs of self-reports were increased in comparison to blood or urine testing (2.93 vs. 2.26) as well as studies in subjects aged under 25 years (3.03) vs. other age groups (2.50). Moreover, five of the nine studies reported co-use of alcohol, but ORs are not reported.

Elvik (7) included the largest research with 27 studies from Europe, USA, Canada, Australia, New Zealand, and Thailand. It received a “high” quality rating (“++”) according to SIGN (23). Study types included were case-control (ten), culpability (nine), epidemiological (seven), and cohort investigations (one). Twenty studies assessed drug use in terms of the results of laboratory analyses of blood or saliva, but it is unclear from the analyses how many studies investigated blood or saliva or other body fluids, like urine.

These studies had a low to moderate methodological quality which was due to differences in controlling for potential confounding variables. Meta-statistics estimated ORs of severity of the crash and computed ORs separately for fatal crashes as well as for injuries and property damage. Cannabis use increased the risk for traffic crash up to 50% while the risk for a fatal crash had an OR of 1.31 (95% CI 0.91–1.88, insignificant, 1.26, 0.88–1.81 after adjusting for publication bias). Car crashes with injuries resulted in an OR of 1.26 (95% CI 0.99–1.6, 1.10, 0.88–1.39) while the risk for crash-related property damage was significantly increased (OR 1.48, 95% CI 1.28–1.72) which remained significant after controlling for publication bias (OR 1.26, 95% CI 1.1–1.44, Table 1). Risk for severe injuries in crashes was higher in studies with self-reported cannabis use (OR = 1.31; 95% CI: 0.8–2.15) vs. laboratory screenings of body fluids (OR = 1.16; 95% CI: 0.79–1.71). In this meta-analysis, again, quality of included studies had a significant effect on outcome ORs. A numerical index of study quality was developed and found that studies with high index scores sometimes reported lower estimates of risk than studies with low index scores (12). It remains unclear which of the studies included in the meta-analysis were of “low” and “high” quality.

Rogeberg and Elvik (17) conducted a meta-analysis on two study samples. The first analysis replicated previous work (2, 16), which the authors found hard to interpret (17).

After re-analysis of the Asbridge study, adjusted OR estimates of 1.25 (95% CI 1.0–1.55) were found (from previously reported 1.95, 1.35–2.73) and for the Li et al. study, adjusted OR was ~1.55 (95% CI 1.10–2.20, from 2.66, 2.07 to 3.41, previously reported).

In the second meta-analysis, 21 observational studies were included. Revised estimates were in a similar range as the adjusted ORs of study 1 (re-analyses of Asbridge et al. and Li et al. data). A statistically significant increase in risk of low-to-moderate magnitude OR 1.36 (1.15–1.61) was reported from the mixed model and an OR of 1.22 (1.1–1.36) in the meta-regression model.

Subsample analyses found relatively higher OR estimates for case–control studies OR 1.36 vs. culpability OR 1.12, low OR 1.45 vs. high quality OR 1.39, no limited control of confounders OR 1.52 vs. high confounder adjustment OR 1.17, and not controlling for alcohol intoxication OR 1.79 vs. controlling OR 1.11. In general, OR estimates of the relationships between cannabis use and car crash risk and subgroups are somewhat lower than those reported from previous meta-analyses (17).

Hostiuc et al. (15) employed two meta-analytical statistical approaches of DUIC and traffic crash risk. These approaches included a random-effects and inverse variance heterogeneity model to assess statistical significance of effect sizes. Altogether, *n* = 24 studies were included into the analyses, of which *n* = 10 relayed on blood analyses (one blood, urine and saliva, two on blood and self-report, one urine and blood, one saliva and blood combinations), *n* = 10 on self-report only (one additional study on self-report and saliva), one on urine analyses and two on “official databases.” Crash risk in DUIC tested via blood analyses had an OR of 1.97, fatal accidents reached an OR of 1.56, and self-reports an OR of 1.94, while the overall effect size for DUIC and crash risk was not statistically significant. Across study types, again, case-control studies presented a higher OR (1.99) than other study types (1.81). Moreover, risk of crashes with injury (OR 2.18) in DUIC and collision (OR 1.81) were higher than those for fatal car crashes (OR 1.73). Using the alternative statistical model, OR estimates were generally 0.2 lower (Table 1). The authors considered several possible causes for their results in the meta-analysis, including heterogeneity of study types and assessment methods. Indeed, no association between DUIC and crash risk and a lack of sensitivity of their statistical approach (random effects model) was found. In addition, publication bias was remarkably high, but rather toward studies reporting a positive association between DUIC and crash risk. To homogenize and focus research on this topic, the authors suggested corroboration with objective data on cannabis use (like blood analyses, with clear cut-off values), or a clinical assessment of the impairment, in the event of a positive screening in traffic, before assessing the individual's fitness to drive (15).

### Relationship Between Different THC Blood Concentrations and Car Vehicle Crashes

Five large case-control studies (26–30) assessed the link between THC blood concentration and crash risk.

Drummer et al. (26) investigated *n* = 3,398 fatal crashes under the influence of alcohol and drugs from three Australian states, including alcohol- and substance-free controls. Using logistic regression analyses; influence of alcohol, cannabis, and other substances; and other factors like age and gender on likelihood of culpability were determined. Drivers with blood THC concentrations of 5 ng/ml or higher showed greater and more statistically significant odds ratio (OR 6.6, 95% CI 1.5–28.0) (Table 2). The estimated odds ratio is greater than that for drivers with a blood alcohol concentration (BAC) of 0.10–0.15% (OR 3.7, 95% CI 1.5–9.1). A significantly positive association with culpability was seen with drivers positive to THC and with BAC ≥ 0.05% compared to those with BAC ≥ 0.05% alone (OR 2.9, 95% CI 1.1–7.7).

**Table 2:** Case control studies of fatal traffic injuries dividing drivers into culpable (or responsible) cases and non-culpable cases.

**References**	**Country**	***N***	**THC in blood ng/ml**	Odds ratio^†^; ^*^ - ^****^	**Confidence interval**	SIGN^****^	**Body tissue or fluid analyzed**
Drummer et al. (30)	Australia	5,000 drivers injured as a result of a vehicular collision	THC (all concentrations)	1.9^****^	1.2–3.1	++	“Blood samples”
			THC 1–4.9	1.6^****^	0.9–2.7		
			THC 5–9.9	1.9^****^	0.7–5.0		
			THC ≥ 5	3.2^****^	1.3–7.2		
			THC ≥ 10	10^****^	1.3–8.2		
Brubacher et al. (29)	Canada	2,318 non-fatally injured motor vehicle drivers	THC = none detected	Reference		++	“Whole blood samples”
			0 < THC < 2	1.09^***^	0.63–1.92		
			2 ≤ THC < 5	1.16^***^	0.66–2.13		
			THC ≥ 5	1.74^***^	0.59–6.36		
			Any THC	1.13	1.03–1.28		
Martin et al. (28)	France	4,059 fatal accidents	Any (≥1)	1.65^**^	1.2–2.3	++	“Blood concentration of THC of over 1 ng/ml”
			THC < 1	1^**^			
			THC 1–3	1.4^**^	0.9–2.1		
			THC 3–5	3.6^**^	1.4–9.5		
			THC ≥ 5	1.6^**^	0.9–3.0		
Laumon et al. (27)	France	10,748 fatal traffic crashes while driving under the influence of cannabis	Any	Reference	1.4–2.3	++	“Blood concentration of THC of over 1 ng/ml”
			THC < 1	1.6^*^	0.8–3.0		
			THC 1–2	1.5^*^	1.1–2.2		
			THC 3–4	2.1^*^	1.2–3.7		
			THC ≥ 5	2.1^*^	1.3–3.4		
			Any dose	1.78^*^	1.4–2.3		
Drummer et al. (26)	Australia	3,398 fatal accidents	Any THC	2.7	1.02–7.0	++	Blood (post mortem)
			THC ≥ 5	6.6	1.5–28.0		

* *Included variables: blood concentration of delta-9-THC, blood concentration of alcohol, age, vehicle type, time of crash*.

** *Included variables: age, gender, vehicle category, time of accident*.

*** *Logistic regression with adjustment for age, sex, health authority, cannabis, alcohol, other recreational drugs, and sedating medications*.

† *Unadjusted model*.

**** *Logistic regression with adjustment for gender, age group, type of vehicle (car/motorbike/heavy vehicle/van or light truck) and location (metro/rural)*.

*SIGN Mythology Checklist: High quality (++), acceptable (+), unacceptable (–), reject (0)*.

The same authors recently conducted a culpability study to include 5,000 injured motor vehicle drivers with comprehensive blood toxicology testing (30). The sample included 1,000 drivers for each of 5 years from ~5,000 to 6,000 drivers injured and taken to hospital in the State of Victoria, Australia. A comprehensive blood-testing was conducted. In a logistic regression, drivers under the influence of THC showed a modest increase in culpability odds, when concentrations of all assessed substances were considered vs. controls (OR 1.9, 95% CI 1.2–3.1, *p* = 0.007). The increase in odds was most obvious at higher blood THC concentrations. At 5 ng/mL and above the OR was 3.2 (*p* = 0.01), and at THC concentrations of 10 ng/mL and above the OR was 10 (*p* = 0.03). At 1–5 ng/ml, the OR was 1.6 (95% CI 0.9–2.7), 5–9 ng/ml 1.9 (95% CI 0.7–5.0), 5 ng/ml and above the OR was 3.2 (95% CI 1.3–7.2, *p* = 0.01), and at THC concentrations of 10 ng/mL and above the OR was 10 (95% CI 1.3–82, *P* = 0.03). These results indicated increasing odds of culpability with rising concentration. THC was the third most prevalent drug detected in 11.1% of all drivers, following alcohol and stimulants. Only 1% of the 5,000 drivers had THC-only at 10 ng/mL or higher. The authors conclude that their culpability analysis of almost 5,000 injured drivers provided further evidence that elevated odds of culpability correlate with increasing THC levels, particularly those with higher blood concentrations.

Laumon et al. (27) conducted a case-control study on cannabis intoxication and fatal road crashes in France. They analyzed *n* = 10,748 drivers, with known drug and alcohol concentrations, involved in fatal crashes in France from October 2001 to September 2003. A total of 6,766 of these were considered culpable at the crash while *n* = 3,306 drivers served as controls. In addition, *n* = 681 drivers were positive for cannabis (cases 8.8%, controls 2.8%), including 285 with an illegal blood alcohol-concentration (≥0.5 g/l). Positive cannabis detection was associated with increased risk of responsibility (OR 3.32, 95% CI 2.63–4.18). A significant dose effect was identified; the odds ratio increased from 2.18 (1.22–3.89) for 0 < THC < 1 ng/ml to THC ≥ 5 ng/ml OR 4.72 (3.04–7.33), uncorrected. The effect of cannabis remains significant after adjustment for different cofactors, including alcohol, with which no statistical interaction was observed (Table 2). At least 2.5% (1.5–3.5%) of fatal crashes were estimated as being attributable to cannabis, compared with 28.6% for alcohol (26.8–30.5%). The authors concluded that driving under the influence of cannabis increases the risk of involvement in a fatal car crash, while the risk in their study was lower than that with positive blood alcohol concentrations.

Martin et al. (28) conducted a similar French cohort study more than a decade later. The authors included *n* = 4,059 drivers. More than 300 of their characteristics, including DUIC and fatal crash risk, were obtained. The proportion of persons driving under the influence of alcohol was 2.1% (95% CI: 1.4 ± 2.8) and under the influence of cannabis 3.4% (2.9–3.9%). Drivers under the influence of cannabis multiplied their risk for causing a fatal crash by 1.65 (1.16 ± 2.34). Strength of the study is certainly its presentation of separate crash ORs for various doses of cannabis detected in blood samples, after adjusting for age, gender, vehicle category, and time of crash, which replicated results from a previous publication of the same research group (27) and the Australian study (26) (Table 2). Another finding of the second French sample is that there was no significant alcohol × cannabis interaction. This means that the increased risk of a fatal crash due to alcohol does not differ significantly from driving under the influence of cannabis (and vice versa).

Brubacher et al. (29) recently conducted a responsibility analysis in a case-control sample to determine whether drivers injured in motor vehicle collisions who tested positive for THC or other drugs are more likely to have contributed to the crash than those who tested negative. Participants included *n* = 2,318 injured drivers who required blood tests for clinical purposes following a motor vehicle collision, of whom 8.3% were screened positively for THC and 14.4% for alcohol. In addition, excess whole blood remaining after clinical use was obtained and broad-spectrum toxicology testing performed. To analyze data, unconditional logistic regression to determine ORs of crash risk for drivers with 0 < THC < 2 ng/ml, 2 ng/ml ≤ THC < 5 ng/ml and THC ≥ 5 ng/ml (all vs. THC = 0 ng/ml). Risk estimates were adjusted for age, gender, and presence of other impairing substances. Odds ratios for all three ranges of blood tested THC were low (Table 2) and not statistically significant. Alcohol × Cannabis interactions were detected for pos. BAC × THC < 2 ng/ml OR = 1.75 (0.37, 17.1) and × THC ≥ 2 ng/ml OR = 1.62 (0.34, 15.7) which did not reach statistical significance. Unadjusted OR for car crash risk under any THC blood level was 1.13 (1.03–1.28). This study, like other culpability and case control studies, relied on blood samples instead of self-reports. Additional strengths are the assessment of alcohol and recreational drug use. However, in comparison to the three previous case-control/culpability studies which included fatal crashes, non-fatal motor crashes with injuries only were analyzed in this study (29).

## Discussion

In this review we first analyzed the general relationship between any crashes in DUIC chronic cannabinoid use, and second, how this relationship changes when potential co-factors are considered. The research on these topics includes five meta-analyses on several study types (case-control, culpability, and cohort studies) (2, 7, 15–17).

All five meta-analyses agreed that cannabinoid use is related to a higher traffic crash risk. However, the OR estimates and 95% CI and statistical significance across studies are variable (Table 1), while the number of included studies unsurprisingly increase over time. The initial meta-analysis (2) analyzed nine studies with *n* = 51,783 participants, the second meta-analysis from the same year (16) also included nine studies with *n* = 92,200 individuals. In this second meta-analysis (16), only two studies (9, 31) were previously analyzed (2). The third meta-analysis (7) with 27 studies included eight studies each of the first (2) and second (16) meta-analysis, while 12 studies were added. Rogeberg and Elvik (17) re-analyzed data from the first and second meta-analysis (2, 16) and added a second study with 21 included investigations, of which *n* = 14 were also analyzed in the previous study of Elvik (7). The most recent analysis (15) included 24 studies, of which 12 were analyzed in the hitherto largest meta-study (7). Thus, there is some, but not complete, overlap of studies included into available meta-analytical research on this topic. Selection criteria also vary across studies. Some include case-control and culpability studies (2, 17) or in addition cohort (16) or laboratory studies (7) and eventually case-control, culpability, survey, and cohort studies (15).

All meta-analyses noted significant heterogeneity across included studies [Asbridge et al. (2): I2: 81; Li et al. (15): heterogeneity: Q = 38.21; *P* < 0.0001; I2 ¼ 79.1; Elvik (7): test for heterogeneity “positive” for all outcomes; Rogeberg and Elvik (17): “there is heterogeneity across studies;” Hostiuc et al. (15): overall Q = 216,59, *p* < 0.001; I2 = 90%]. The last study also presented heterogeneity statistics for several outcome criteria [collision subgroup Q = 47,06, *p* < 0.001, I2 89%; injury subgroup Q = 62.0; *p* < 0.001, I2 82%; fatal Q = 19.26, *p* < 0.001, I2 79% (15)].

All these factors may explain why there is some variation in the OR estimates across meta-analyses for the overall statistics. Further, case-control studies reach higher OR estimates than culpability studies and, except for (2), fatal crashes lower OR estimates than other kinds of crashes. Finally, studies based on self-reports have higher ORs than those based on blood or urine results. Culpability studies (2) include drivers involved in collisions, sub-grouped into those responsible for the collision and those not responsible. The premise of these studies is, if cannabis use increases collision risk, then it should more likely be detected in drivers judged to be responsible for their collision. However, OR may be higher in case-control studies, where DUIC subjects are compared to non-DUIC controls. Further, DUIC may impair driving skills and increase crash risk, however, risk of fatal crashes is lower than other types of crashes (collision only, injury). Thus, the increased risk for car crashes is relatively higher for collision and injury and somewhat attenuated but still increased for fatalities.

Self-reports of cannabis use may be subject to recall bias and may be less precise to detect actual cannabis use compared to blood or urine tests. Usually, it is assumed that cannabis use is underestimated in self-reports. As commented in previous analyses (16) different methods of assessing cannabis use (e.g., self-report, urine tests, and blood tests) may have different levels of validity and reliability. The authors of one meta-analysis (17) stated that, to their view, laboratory analyses of blood samples for all subjects included in a study provide the best information on acute intoxication while driving. The second best indicator is saliva. Urine is a less informative indica tor, as inactive metabolites of cannabis can be detected in samples of urine a long time after the substance became inactive.

Most of these screenings determine whether cannabis was used within the past few weeks, whereas acute impairment in driving skills from cannabis use lasts between 3 and 12 h (16, 33). Cannabis being an illicit drug in most countries, drivers in the comparison groups might be less likely than those involved in crashes to submit to testing, which could lead to overestimation of the effect of marijuana use on crash risk.

These meta-analyses have several limitations. The fourth meta-analysis (17) found only five studies that calculated crash risk for drivers with blood THC > 2 ng/ml. Further, all case–control studies had high refusal rates (>15%), potentially resulting in selection bias if drivers who refused participation had different rates of drug use than those who participated. Different methods to detect cannabis exposure in cases vs. controls (e.g., blood THC in cases and saliva THC in controls) were employed in many case-control studies, non-comparable controls (e.g., patients visiting hospital for medical problems) were recruited to estimate THC use in the general driving population (29), and only a fraction of included studies measured THC in blood samples or a combination of blood samples and urine or saliva samples or self-reports [Asbridge et al. 9 of 10 (2), Elvik 20 of 27 (7), Li et al. 2 of 9 (16), and Hostiuc et al. 10 of 24 studies (15)]. All of these limit the generalizability of findings.

In addition, whole blood, plasma, urine, and saliva are body fluids with different characteristics when it comes to assessment of THC levels. Experimental studies could not find a significant correlation between THC concentrations (logarithms) in oral fluid and plasma, while time influences THC concentrations in plasma and oral fluid differently after repeated oral THC doses (33). Cannabinoids in urine have a longer window of detection than blood and oral fluids, and THC's elimination is non-linear so that reverse extrapolation of concentrations to an earlier time is not feasible. When utilizing blood rather than plasma cannabinoid concentrations, concentrations must be approximately doubled because cannabinoids are tightly bound to proteins in plasma (>90%), and there is minimal partitioning into erythrocytes (34).

Also, cannabinoid blood and plasma concentrations were significantly higher in frequent smokers compared with occasional smokers at most time points for THC and 11-OH-THC (35). In oral fluids, THC levels above 2 μg/L were detected in occasional smokers for 26 h while frequent smokers had higher levels for >72 h. Thus, low THC concentrations can be detected for several days in oral fluids of chronic smokers similar to blood and urine (36). Eventually, the authors of the review comment that cannabinoid stability in these measures not only depend on characteristics of the body fluids used and the smoking status of the individuals, it also depends on collection method, buffer composition in commercial collection devices, the analytes, storage containers, and storage temperature and duration.

The third purpose of our analyses on the relationship between THC concentration and car crash risk was addressed by five case-control studies with “high quality (++)” SIGN rating (Table 2). None of the meta-analyses reported statistics on this relationship. However, the association is of importance since there are various legal cut-off values for THC blood levels across European countries for DUIC. While the penalty increases in Norway according to the THC concentration detected (1.3, 3, 9 ng/ml), other countries have cut-off values of 1 ng/ml (Germany, Belgium, Ireland, Luxemburg, Netherlands in the presence of other substances), 2 ng/ml (Czech Republic, United Kingdom), and 3 ng/ml (Netherlands) [EMCDDA Cannabis and Driving (5)]. It is a relevant issue whether studies report evidence using which of the legal cut-off concentrations supported by empirical data.

The Australian study (26) indicated a significantly higher risk for THC ≥ 5 ng/ml with the highest OR estimates across studies (OR 6.6) which is higher than ORs from later studies [Laumon et al. (27) unadjusted OR (uaOR) 4.7, Martin et al. (28) (uaOR) 3.95, and Brubacher et al. (29) (uaOR) 2.29 for the same subgroup of individuals with THC ≥ 5 ng/ml]. However, in the Drummer et al. sample, in 84% of the THC-only cases the THC concentration was ≥5 ng/ml and the median was 12 ng/ml. This rate is higher compared to the French and Canadian samples [Laumon et al. (27): 2.66%, Martin et al. (28): 4.2%, Brubacher et al. (29): 0.9%]. Therefore, based on the high rate of individuals with THC above 5 ng/ml, it was quite likely to see an effect of THC on crash risk in the Australian sample. Further, in the Australian study (26), alcohol was commonly found in THC-positive cases (43%), the effect of THC was also evaluated in the THC plus alcohol cases and significant interaction was found. This interaction could not be replicated in French and Canadian samples. In the first French study (27), no statistical interaction between blood concentrations of THC and alcohol was detected as well as in the later studies (28, 29). Thus, most studies report a significant influence of cannabis and alcohol use on culpability for fatal crashes or injuries, but both substances obviously exert their effects independently (6). Results from the Australian Study (26) also suggested that both alcohol and cannabis showed a biological gradient with higher doses of both substances have a higher OR of culpability in fatal crashes. This significant dose-effect was also reported from the first French (27) and the Canadian Study (29), while in the second French study (28), the ORs show an inverse U-curve trend with a maximum at THC concentrations between >2 and <5 ng/ml, after controlling for alcohol intoxication. All authors across studies consistently argue that their studies yield a marked dose effect and a potential causal role of DUIC in fatal crashes. The latest study of Drummer et al. (30) confirmed the finding that elevated odds of culpability are positively associated with THC, particularly those with higher blood concentrations.

Culpability studies may be well suited to compare risk of fatal and non-fatal crashes, because THC blood levels were measured in all five samples. If THC blood levels are compared regarding fatal (26–28) and non-fatal crashes (29, 30), the odds ratios for any THC blood levels are similar [fatal crashes: 2.7 ng/ml (26), 1.78 ng/ml (27), 1.65 ng/ml (28) vs. non-fatal crashes 1.13 ng/ml (29), 1.9 ng/ml (30)] while the levels in fatal crashes tended to be higher. A previous meta-analysis and systematic review, which excluded low-quality studies, reported cannabis-associated risk for non-fatal crashes (OR = 1.74; 95% CI = 0.88–3.46) and for fatal crashes (OR = 2.1; 95% CI = 1.31–3.36) (2) and confirm the findings for the included five culpability studies in our analysis.

However, case-control and culpability studies have limitations. As several of the authors pointed out, estimating the degree of intoxication for cannabis is a more difficult task than for alcohol (26, 29). As far as cannabis is concerned, after smoking and vaporization, there is a delay in maximal cannabis effects compared to the peak THC blood concentration after consumption, with peak effects after ~30 min depending upon study design and time of testing. Effects on cognition and psychomotor function do not decline as quickly as THC concentration decreases (37–40). When smoking a “joint,” whole blood THC concentrations typically peak at >100 ng/ml during smoking and then drop so rapidly that THC is usually <2 ng/ml within 4 h of a single acute exposure (38). Psychotropic effects typically peak at 20–30 min and resolve by 4 h. Ingesting cannabis delays the onset and extends the duration of these effects. The main THC metabolite, 11-nor-9-carboxy-THC (THC-COOH), is not psychoactive and persists in blood and urine long after impairment resolves. Thus, THC-COOH provides evidence of previous cannabis exposure but does not necessarily indicate impairment or recent use (41). The active ingredient behind most of the effects of cannabis that impair driving ability is THC. Metabolites such as THC-COOH are present and detectable for a significant time after consumption but lack any proven psychoactive effects which impair driving ability.

The results of culpability and case-control studies must be compared with findings from experimental and laboratory studies. Laboratory and simulator studies certainly suffer from the limitation that they are conducted in an artificial environment. Thus, experimental data show that drivers attempt to compensate cannabis intoxication by slower driving after smoking cannabis, but their control deteriorates with increasing task complexity (5, 6). These behaviors limit the generalizability of experimental study results to authentic traffic situations (2). However, as with other cognitive tasks, cannabis smoking (THC-dominant and THC-CBD-equivalent) increases lane weaving after 40–100 min following vaporization (9) and impaired cognitive function consistently as well as critical-tracking tasks, reaction times, divided-attention tasks, and lane-position variability, all of which may increase car crash risk.

In general, however, there is no clear overall relationship with THC blood or serum levels and driving skills or crash risk from experimental studies, even if time of use and duration of consumption are considered. Not surprisingly, there is no unanimous agreement on potential THC legal cut-off levels despite the dose effect with higher THC blood concentrations resulting in higher OR estimates for crashes. However, a relationship does not necessarily provide a clue for a scientifically supported cut-off value. Experts suggest that many drivers with blood THC >3 ng/ml (42) or >3–5 ng/ml (43) have significant impairment and should be prohibited from driving. Based on these reports, many jurisdictions, including many US states and Canada, have set THC *per se* limits of 2 or 5 ng/ml, while many European countries have a limit of 1 ng/ml. Advocates of these lower THC concentrations argue that THC concentration drops rapidly after smoking, so a driver impaired with high THC concentrations at the time of driving could show concentrations below 5 ng/ml several hours later if there is a delay in obtaining blood samples (44), a fact that supports lower *per se* limits for THC (30). These concentrations, especially the 1 or 2 ng/ml levels, were criticized because they may not indicate impairment especially in frequent users who develop tolerance to some THC impairing effects (6, 42). Due to the accumulation of cannabinoids in fat, some daily users may have blood THC > 1 ng/ml after a week or more of abstinence (6). Therefore, the various THC concentrations used to define a cannabis-related driving offense in EU countries and some US-states varying between 1 and up to 7 ng/ml alone may not be appropriate to evaluate driving skill impairment comprehensively.

Measures of THC and its non-psychoactive metabolite THC-COOH within hours of an accident suffer from several limitations and may not reflect driving skill impairment. Even higher THC concentrations of >5 ng/ml in recent studies yielded higher risks of injuries and fatal concentrations, however not all studies reported statistically significant relationships. Rather, the assessment of both biological measures and psychomotor characteristics may render evaluation of driving fitness more accurate (more valid to “real” impairment) and is probably applicable in practical traffic control situations. Thus, future experimental and laboratory research on cannabinoid's effects on fitness to drive should consider designs which include measures of psychomotor and cognitive functions like reaction times and decision making after long and monotonous drives and divided attention tasks. These cognitive characteristics may be most impaired by cannabis according to available research (5, 6).

Finally, it is important to recognize that an important compound of cannabis-products used is Cannabidiol (CBD). As for driving skills, it is a substance with sedative effects and therefore may play a role in impairing driving abilities and increase risks for accidents. However, no study investigates the influence of CBD alone on car crash risks. Certainly, since CBD is in increasing demand in sales and consumption, further studies are needed to evaluate the relationship between CBD use, driving ability, and car crash risks.

In summary, there is unanimous agreement across studies that acute cannabis use significantly increases the risk for car crashes and impairs specific driving skills, and confidence in the results from several types of studies (case-control, culpability, and cohorts) is rated “moderate” according to CERQual. In meta-analyses, cannabis user's ORs for car crashes are slightly but significantly increased, when several confounders are considered in multivariate analyses. Further, self-report vs. blood test, case control vs. culpability, and non-fatal crashes had higher OR estimates. High quality culpability studies (SIGN) noted that there is a dose effect of higher THC blood concentrations with increased risk for fatal crashes and those with injuries. However, this biological gradient does not provide a clear legal cut off value. Therefore, these cut off values still range between 1 and 5 ng/ml. While there are various problems related to the measure of THC and its metabolite THC-COOH in blood and other tissues within hours after a crash (different body fluids with different time frames of THC levels), these values may not even reflect actual driving skill impairment. Thus, the biological measures alone may be not appropriate to assess fitness to drive and should be combined with ratings of psychomotor and cognitive skills.

## Data Availability Statement

The original contributions presented in the study are included in the article/supplementary material, further inquiries can be directed to the corresponding author/s.

## Author Contributions

UP, MH, DH, JW, and EH wrote, edited, and supervised the manuscript. MS, BL, and AH contributed on the CAPRIS study by conducting the literature search and evaluating it. All authors contributed to the article and approved the submitted version.

## Conflict of Interest

The authors declare that the research was conducted in the absence of any commercial or financial relationships that could be construed as a potential conflict of interest.

## References

[1] World Drug Report. United Nations Publication, Sales No. E.18.XI.9 (2018).

[2] Asbridge M Hayden JA Cartwright JL. Acute cannabis consumption and motor vehicle collision risk: systematic review of observational studies and meta-analysis. Br Med J. (2012) 344:e536. 10.1136/bmj.e536 22323502 PMC3277079

[3] European Monitoring Centre for Drugs Drug Addiction EMCDDA. Driving Under the Influence of Drugs, Alcohol and Medicines in Europe — Findings From the DRUID Project. Luxembourg: Publications Office of the European Union (2012).

[4] Compton R. Marijuana-Impaired Driving: A Report to Congress, National Highway Safety Transport Administration. Washington, DC (2017). Available online at: https://www.nhtsa.gov/sites/nhtsa.dot.gov/files/documents/812440-marijuana-impaired-driving-report-to-congress.pdf (accessed November 28, 2020).

[5] European Monitoring Centre for Drugs Drug Addiction EMCDDA. Cannabis and Driving. Question and Answers for Policy Making. Lisbon (2018).

[6] Hartman RL Brown TL Milavetz G Spurgin A Gorelick DA Gaffney G. Controlled cannabis vaporizer administration: blood and plasma cannabinoids with and without alcohol. Clin Chem. (2015) 61:850–69. 10.1373/clinchem.2015.238287 26019183

[7] Elvik R. Risk of road accident associated with the use of drugs: a systematic review and meta-analysis of evidence from epidemiological studies. Accident Anal Prevent. (2013) 60:254–67. 10.1016/j.aap.2012.06.017 22785089

[8] Ramaekers JG Berghaus G van Laar M Drummer OH. Dose related risk of motor vehicle crashes after cannabis use. Drug Alcohol Dependence. (2004) 73:109–19. 10.1016/j.drugalcdep.2003.10.008 14725950

[9] Arkell TR Vinckenbosch F Kevin RC Theunissen EL McGregor IS Ramaekers JG. Effect of cannabidiol and Δ9-tetrahydrocannabinol on driving performance: a randomized clinical trial. JAMA. (2020) 324:2177–86. 10.1001/jama.2020.21218 33258890 PMC7709000

[10] Blows S Ivers RQ Connor J Ameratunga S Woodward M Norton R. Marijuana use and car crash injury. Addiction. (2005) 100:605–11. 10.1111/j.1360-0443.2005.01100.x 15847617

[11] Mann RE Adlaf E Zhao J Stoduto G Ialomiteanu A Smart RG. Cannabis use and self-reported collisions in a representative sample of adult drivers. J Saf Res. (2007) 38:669–74. 10.1016/j.jsr.2007.09.004 18054598

[12] Terry-McElrath YM O'Malley PM Johnston LD. Alcohol and marijuana use patterns associated with unsafe driving among U.S. high school seniors: high use frequency, concurrent use, and simultaneous use. J Stud Alcohol Drugs. (2014) 75:378–89. 10.15288/jsad.2014.75.378 24766749 PMC4002852

[13] Gerberich SG Sidney S Braun BL Tekawa IS Tolan KK Quesenberry CP. Marijuana use and injury events resulting in hospitalization. Ann Epidemiol. (2003) 13:230–7. 10.1016/S1047-2797(02)00411-8 12684188

[14] Asbridge M Mann R Cusimano MD Trayling C Roerecke M Tallon JM. Cannabis and traffic collision risk: findings from a case-crossover study of injured drivers presenting to emergency departments. Int J Public Health. (2014) 59:395–404. 10.1007/s00038-013-0512-z 24061594

[15] Hostiuc S Moldoveanu A Negoi I Drima E. The association of unfavorable traffic events and cannabis usage: a meta-analysis. Front Pharmacol. (2018) 9:99. 10.3389/fphar.2018.00099 29487531 PMC5816577

[16] Li MC Brady JE DiMaggio CJ Lusardi AR Tzong KY Li. Marijuana use and motor vehicle crashes. Epidemiol Rev. (2012) 34:65–72. 10.1093/epirev/mxr017 21976636 PMC3276316

[17] Rogeberg O Elvik R. The effects of cannabis intoxication on motor vehicle collision revisited and revised. Addiction. (2016) 111:1348–59. 10.1111/add.13347 26878835

[18] Hoch E Friemel C Schneider M. Cannabis: Potenzial und Risiko. Eine wissenschaftliche Bestandsaufnahme. Heidelberg: Springer (2018).

[19] Higgins J Green S. Cochrane Handbook for Systematic Reviews of Interventions, vol Version 5.1.0. London: The Cochrane Collaboration (2011).

[20] Hoch E Friemel C Schneider M Pogarell O Hasan A Preuss UW. CaPRis-Projektgruppe Efficacy and safety of medicinal cannabis: results of the CaPRis study. Bundesgesundheitsblatt Gesundheitsforschung Gesundheitsschutz. (2019) 62:825–9. 10.1007/s00103-019-02965-3 31214723

[21] AWMF (Arbeitsgemeinschaft Wissenschaftlich Medizinischer Fachgemeinschaften). Manual systematische Recherche für Evidenzsynthesen und Leitlinien. (2019). Available online at: https://www.awmf.org/fileadmin/user_upload/Leitlinien/Werkzeuge/20190403_Manual_Recherche.pdf (accessed November 15, 2020).

[22] Liberati A Altman DG Tetzlaff J Mulrow C Gøtzsche PC Ioannidis JPA. The PRISMA statement for reporting systematic reviews and meta-analyses of studies that evaluate health care interventions: explanation and elaboration. PLOS Med. (2009) 6:e1000100. 10.1371/journal.pmed.1000100 19621070 PMC2707010

[23] Network SIG. SIGN 50 Methodology Checklist. Edinburgh (2015).

[24] OCEBM: Levels of Evidence Working Group. The Oxford Levels of Evidence 2. 2nd ed. Oxford Centre for Evidence-Based Medicine (2011). Available online at: http://www.cebm.net/index.aspx?o=5653 (accessed May 5, 2021).

[25] Girotto E Mesas AE deAndrade SM Birolim MM. Psychoactive substance use by truck drivers: a systematic review. Occupat Environ Med. (2014) 71:71–6. 10.1136/oemed-2013-101452 24145953 PMC3888602

[26] Drummer OH Gerostamoulos J Batziris H Chu M Caplehorn J Robertson MD. The involvement of drugs in drivers of motor vehicles killed in Australian road traffic crashes. Accid Anal Prev. (2004) 36:239–48. 10.1016/S0001-4575(02)00153-7 14642878

[27] Laumon B Gadegbeku B Martin JL Biecheler MB the SAM Group. Cannabis intoxication and fatal road crashes in France: population-based case-control study. BMJ. (2005) 331:1371. 10.1136/bmj.38648.617986.1F 16321993 PMC1309644

[28] Martin JL Gadegbeku B Wu D Viallon V Laumon B. Cannabis, alcohol and fatal road accidents. PLoS ONE. (2017) 12:e0187320. 10.1371/journal.pone.0187320 29117206 PMC5678710

[29] Brubacher JR Chan H Erdelyi S Macdonald S Asbridge M Mann RE. Cannabis use as a risk factor for causing motor vehicle crashes: a prospective study. Addiction. (2019) 114:1616–26. 10.1111/add.14663 31106494 PMC6771478

[30] Drummer OH Gerostamoulos D Di Rago M Woodford NW Morris C Frederiksen T. Odds of culpability associated with use of impairing drugs in injured drivers in Victoria, Australia. Accid Anal Prev. (2020) 135:105389. 10.1016/j.aap.2019.105389 31812899

[31] Peterson J Welch V Losos M Tugwell P. The Newcastle-Ottawa Scale (NOS) for Assessing the Quality of Nonrandomised Studies in Meta-analyses. (2011). Available online at: http://www.ohri.ca/programs/clinical_epidemiology/oxford.asp (accessed July 28, 2019).

[32] Woratanarat P Ingsathit A Suriyawongpaisal P Rattanasiri S Chatchaipun P Wattayakorn K. Alcohol, illicit and non-illicit psychoactive drug use and road traffic injury in Thailand: a case-control study. Accid Anal Prevent. (2009) 41:651–7. 10.1016/j.aap.2009.03.002 19393818

[33] Huestis MA Sempio C Newmeyer MN Andersson M Barnes AJ Abulseoud OA. Free and glucuronide urine cannabinoids after controlled smoked, vaporized and oral cannabis administration in frequent and occasional cannabis users. J Anal Toxicol. (2020) 44:651–60. 10.1093/jat/bkaa046 32369162 PMC7673603

[34] Milman G Schwope DM Schwilke EW Darwin WD Kelly DL Goodwin RS. Oral fluid and plasma cannabinoid ratios after around-the-clock controlled oral Δ(9)-tetrahydrocannabinol administration. Clin Chem. (2011) 57:1597–606. 10.1373/clinchem.2011.169490 21875944 PMC3836268

[35] Karschner EL Swortwood-Gates MJ Huestis MA. Identifying and quantifying cannabinoids in biological matrices in the medical and legal cannabis era. Clin Chem. (2020) 66:888–914. 10.1093/clinchem/hvaa113 32628766

[36] Desrosiers NA Himes SK Scheidweiler KB Concheiro-Guisan M Gorelick DA Huestis MA. Phase I and II cannabinoid disposition in blood and plasma of occasional and frequent smokers following controlled smoked cannabis. Clin Chem. (2014) 60:631–43. 10.1373/clinchem.2013.216507 24563491

[37] Desrosiers NA Huestis MA. Oral fluid drug testing: analytical approaches, issues and interpretation of results. J Anal Toxicol. (2019) 43:415–43. 10.1093/jat/bkz048 31263897

[38] Hunault CC Bocker KB Stellato RK Kenemans JL deVries I Meulenbelt J. Acute subjective effects after smoking joints containing up to 69 mg Delta9-tetrahydrocannabinol in recreational users: a randomized, crossover clinical trial. Psychopharmacology. (2014) 231:4723–33. 10.1007/s00213-014-3630-2 24879495

[39] Spindle TR Cone EJ Schlienz NJ Mitchell JM Bigelow GE Flegel R. Acute pharmacokinetic profile of smoked and vaporized cannabis in human blood and oral fluid. J Anal Toxicol. (2019) 43:233–58. 10.1093/jat/bky104 30615181 PMC6676961

[40] Battistella G Fornari E Thomas A Mall JF Chtioui H Appenzeller M. ‘Weed or wheel! FMRI, behavioural, and toxicological investigations of how cannabis smoking affects skills necessary for driving.’ PLoS ONE. (2013) 8:e52545. 10.1371/journal.pone.0052545 23300977 PMC3534702

[41] Huestis MA Barnes A Smith ML. Estimating the time of last cannabis use from plasma delta9- tetrahydrocannabinol and 11-nor-9-carboxy-delta9-tetrahydrocannabinol concentrations. Clin Chem. (2005) 51:2289–95. 10.1373/clinchem.2005.056838 16223887

[42] Castillo C. Drink and drug driving policy in the United Kingdom: assessing impact. In: Third International Symposium on Drug-Impaired Driving, European Monitoring Centre for Drugs and Drug Addiction. Lisbon (2017).

[43] Liguori A Gatto CP Robinson JH. Effects of marijuana on equilibrium, psychomotor performance, and simulated driving. Behav Pharmacol. (1998) 9:599–609. 10.1097/00008877-199811000-00015 9862085

[44] McClure E Stitzer M Vandrey R. Characterizing smoking topography of cannabis in heavy users. Psychopharmacology. (2012) 220:309–18. 10.1007/s00213-011-2480-4 21922170 PMC3641906

